# Supplementary material for: Temporal Mortality Trends Attributable to Stroke in South Asia: An Age–Period–Cohort Analysis
Source: Healthcare (Basel). 2024 Sep 10;12(18):1809. doi: 10.3390/healthcare12181809 (PMC11430981; doi:10.3390/healthcare12181809)
Supplement: Supplementary file 1 [file healthcare-12-01809-s001.zip › healthcare-3054139-Supplementary Materials.pdf]

Appendix: Temporal mortality trends attributable to stroke in South Asia: an age-period-cohort analysis

Content

Figure S1: Age-Standardized Mortality of Intracerebral Hemorrhage for Four South Asian Countries 2

Figure S2: Age-Standardized Mortality of Ischemic Stroke for Four South Asian Countries 3

Figure S3: Age-Standardized Mortality of Subarachnoid Hemorrhage for Four South Asian Countries 4

Table S1. Crude Mortality Rate of Stroke Deaths by Age, Period, and Median Birth Cohorts in South Asia, 1990 to 2019 (per 100,000) 5

Table S2: Number of Stroke Deaths by Age, Period, and Median Birth Cohorts in South Asia, 1990 to 2019 10

Table S3: Estimated Longitudinal Age and Cross-Sectional Age-Specific Trends in Stroke for the Periods 1990–1994 and 2015–2019 15

Table S4: Annual Percentage Change for Stroke Mortality Overall and in Each Age Group Across Four South Asian Countries from 1990 to 2019 16

Table S5: Fitted Longitudinal Age Effects of Stroke Mortality (per 100,000 person-years) and the Corresponding 95% CIs 18

Table S6: Relative Risk of Each Period Compared with the Reference (2000–2004) and the Corresponding 95% CIs 20

Table S7: Relative Risk of Each Cohort Compared with the Reference (Cohort 1955–1959) and the Corresponding 95% CIs 21

Figure S1: Age-Standardized Mortality of Intracerebral Hemorrhage for Four South Asian Countries

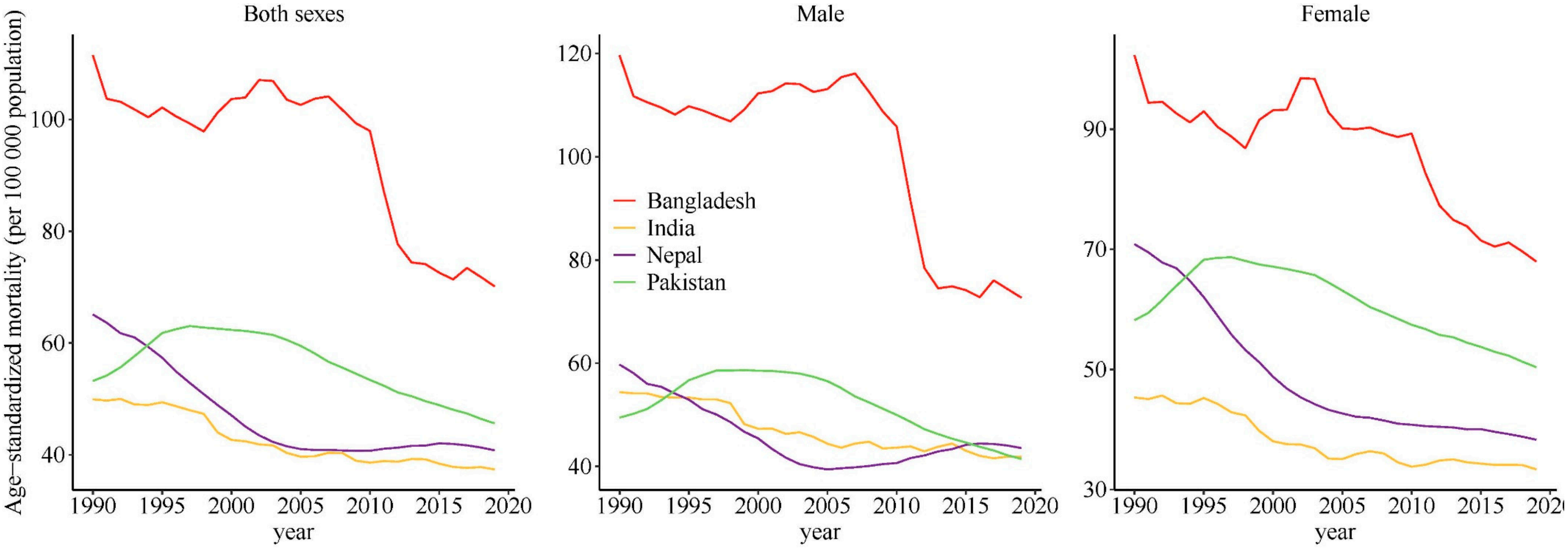

The data of this figure was extracted from: https://vizhub.healthdata.org/gbd-results/

Figure S2: Age-Standardized Mortality of Ischemic Stroke for Four South Asian Countries

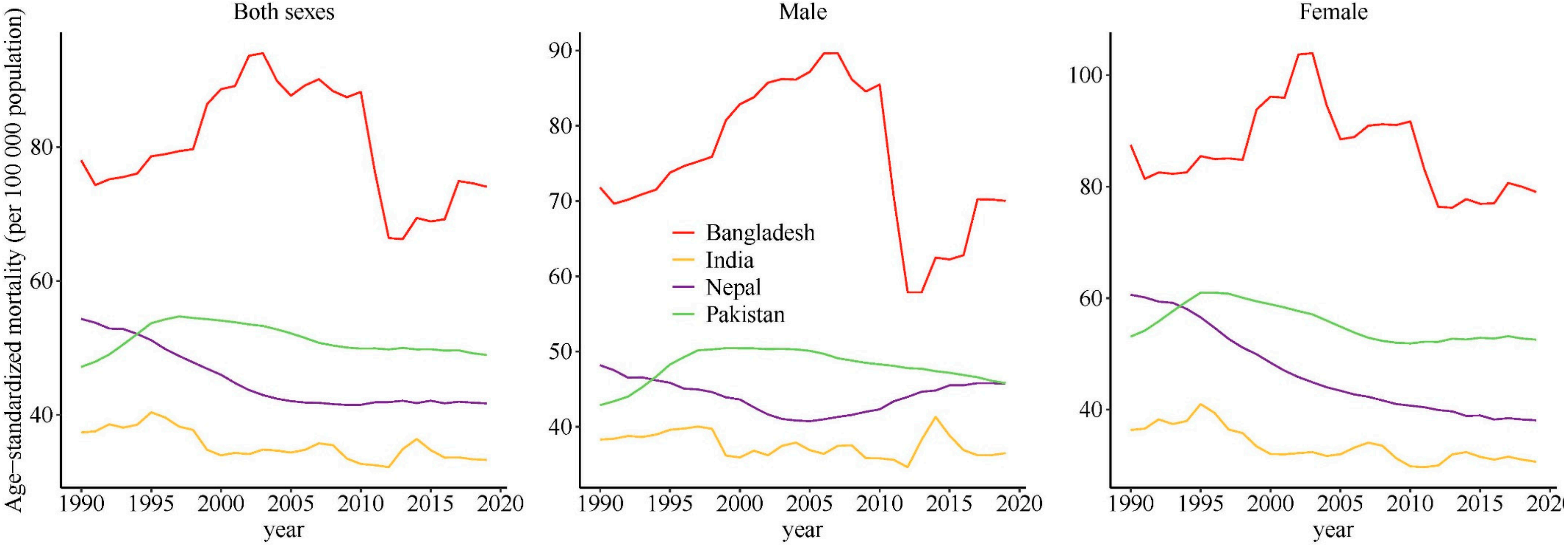

The data of this figure was extracted from: https://vizhub.healthdata.org/gbd-results/

Figure S3: Age-Standardized Mortality of Subarachnoid Hemorrhage for Four South Asian Countries

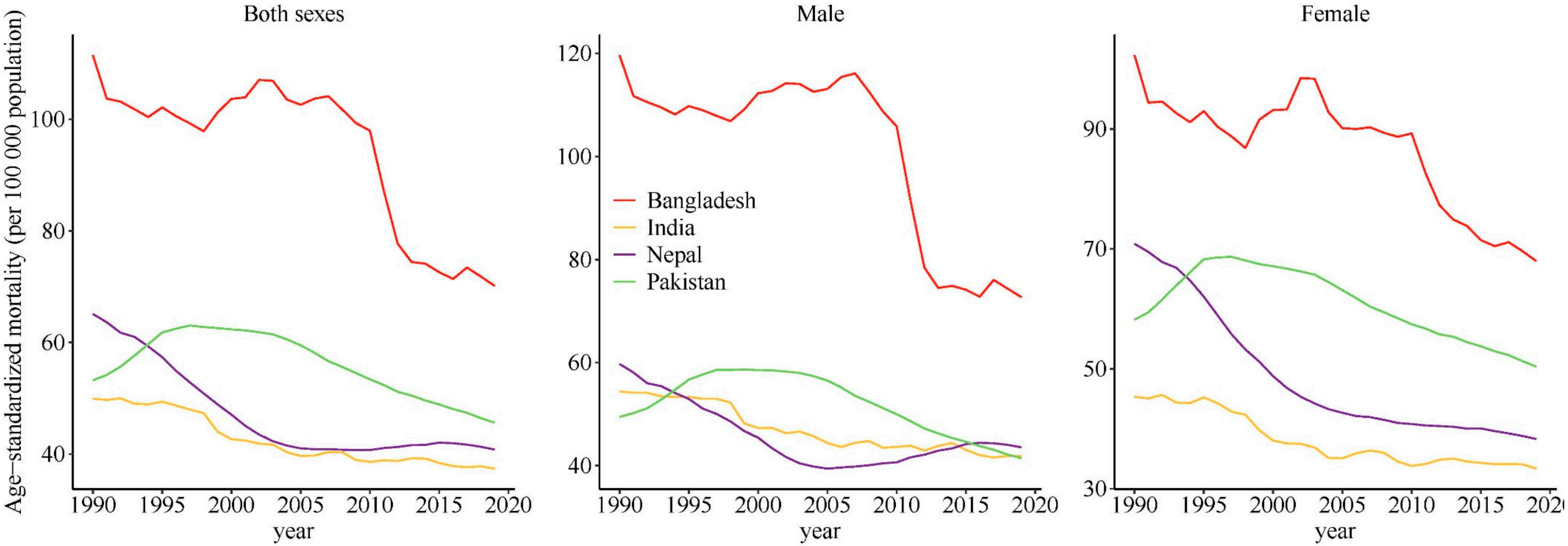

The data of this figure was extracted from: https://vizhub.healthdata.org/gbd-results/

Table S1. Crude Mortality Rate of Stroke Deaths by Age, Period, and Median Birth Cohorts in South Asia, 1990 to 2019 (per 100,000)

Median Period	Age group															Median Birth Cohort
	15-19	20-24	25-29	30-34	35-39	40-44	45-49	50-54	55-59	60-64	65-69	70-74	75-79	80-84	85-89	
Bangladesh															3381.1	1905
														2143.2	3567	1910
													1475.9	2223.4	5098.3	1915
												843.8	1426	3097.8	5555.8	1920
											600.6	849.4	1567.5	3496.6	3396.8	1925
										479.8	615	995.4	1654.9	2133.3	3549.3	1930
									191.2	439.3	654.2	1102.4	1280.4	2160.4		1935
								159.9	184.6	456.4	706.8	939.8	1186			1940
							70.7	151.3	189.9	457.7	534	790.6				1945
						47.7	66.7	143.5	204.5	382.6	504.2					1950
					24.3	41.3	63.3	141.1	180.5	314.5						1955
				15.3	22	37	59.7	132.6	153							1960
			9.1	13.7	21.2	36.9	58.8	108.9								1965
		5	7.9	12.8	20.9	30.5	48.4									1970
	2.9	4.3	6.5	13.1	15.8	27.8										1975
1992	2.4	4.2	6.3	9.7	15.8											1980
1997	2.4	4.2	4.8	10.1												1985
2002	2.4	3.5	5.7													1990
2007	1.7	3.7														1995
2012	1.7															2000
2017																

Median Period	Age group															Median Birth Cohort
	15-19	20-24	25-29	30-34	35-39	40-44	45-49	50-54	55-59	60-64	65-69	70-74	75-79	80-84	85-89	
India															1890.7	1905
														1245.7	1896	1910
													1094.3	1221.3	1804.8	1915
												692.2	1056.3	1150.8	1612.1	1920
											473.2	676	1000.7	1012.9	1045.8	1925
										283.3	455.9	631.4	854.4	726.2	1028.6	1930
									149.4	278.5	419.4	539.6	671	842.7		1935
								80.6	144.2	218.6	359.8	493.9	623.7			1940
							40.3	86.5	128.5	216.2	354.6	433.2				1945
						19	39.5	67.4	107.3	205.6	295.8					1950
					9.7	19.4	35.2	60.9	114.7	188.9						1955
				6	9.5	17.1	33.2	62.6	114.6							1960
			3.2	6.2	10.5	18.4	34.4	65.2								1965
		2.7	3.4	5.7	9.8	17.6	29.5									1970
	1.6	2.6	3.5	5.3	9.7	16.1										1975
1992	1.3	2.4	2.9	4.7	8.1											1980
1997	1.3	2.4	2.7	4												1985
2002	1.2	2.1	1.9													1990
2007	1	1.4														1995
2012	0.6															2000
2017																

Median Period	Age group															Median Birth Cohort
	15-19	20-24	25-29	30-34	35-39	40-44	45-49	50-54	55-59	60-64	65-69	70-74	75-79	80-84	85-89	
Nepal															1677.8	1905
														1189	1575	1910
													1031.6	1103.1	1444.7	1915
												667	941.5	992.3	1409.7	1920
											433.8	603.1	843.6	967.1	1497.2	1925
										277.7	383.9	530	807.8	993.6	1606.2	1930
									156.9	240.5	324.7	510.7	814.2	989.7		1935
								92.5	132.1	197.7	309.5	513.7	824.3			1940
							46.8	76.3	104.9	188.1	313.7	511.9				1945
						24.3	38.3	59.1	99.3	188	315					1950
					11	20.2	29.4	55.4	101.5	188.3						1955
				5.7	9.3	15.3	26.8	56.4	100.5							1960
			3	4.9	7.2	14.2	27.2	54.7								1965
		1.8	2.5	3.8	6.5	14.2	26.4									1970
	0.9	1.6	2	3.3	6.4	13.1										1975
1992	0.8	1.3	1.7	3.1	5.8											1980
1997	0.7	1.3	1.6	2.8												1985
2002	0.7	1.3	1.5													1990
2007	0.7	1.3														1995
2012	0.8															2000
2017																

Median Period	15-19	20-24	25-29	30-34	35-39	40-44	45-49	50-54	55-59	60-64	65-69	70-74	75-79	80-84	85-89	Median Birth Cohort
Pakistan															2184.1	1905
														1502.8	2326.8	1910
													1253.8	1597.4	2297.3	1915
												829.9	1364.9	1552.1	2210.6	1920
											528.1	914.3	1331.7	1489.4	2109.5	1925
										332	601.2	888	1256.9	1435.9	1978.1	1930
									188.2	380.7	589.4	832.7	1188.4	1362.9		1935
								111.9	220.2	373.9	545.1	789.8	1115.2			1940
							58	131.9	214.8	343.4	505.2	751.5				1945
						31.4	68.8	128.5	195	317.5	474.2					1950
					15.2	37.4	67.5	116	177.3	297.8						1955
				9.4	18	37.4	61.2	105	164							1960
			4.6	11.2	18.3	34.6	55.6	96.9								1965
		2.9	5.5	11.5	17.3	32.2	51.7									1970
	1.7	3.6	5.6	11.2	16.3	30.4										1975
1992	2.1	3.9	5.5	10.6	15.8											1980
1997	2.3	3.9	5.4	10.3												1985
2002	2.3	3.8	5.4													1990
2007	2.3	3.9														1995
2012	2.4															2000
2017																

Median Period	Age group															Median Birth Cohort
	15-19	20-24	25-29	30-34	35-39	40-44	45-49	50-54	55-59	60-64	65-69	70-74	75-79	80-84	85-89	
South Asia															2179.1	1905
														1383.5	2189.4	1910
													1152.9	1385.9	2304.9	1915
												719.4	1118.6	1375.1	2048.3	1920
											486.4	709.2	1070	1254.7	1333.2	1925
										301.7	478	679.9	955.5	915.9	1381.1	1930
									155.8	297	447.3	605.8	764.1	1017.1		1935
								89.6	153.7	247.8	398.9	553.8	714			1940
							44.3	95.6	140.7	244.4	379.9	490.2				1945
						22.5	44.3	79.1	122.7	228.1	326.6					1950
					11.5	22.8	40.3	72.7	125.6	208.3						1955
				7.2	11.3	20.6	37.9	72.5	122.2							1960
			3.9	7.4	12.1	21.4	38.4	71.9								1965
		2.9	4.1	6.9	11.4	20.0	33.2									1970
	1.7	2.9	4.0	6.6	10.9	18.5										1975
1992	1.5	2.7	3.5	5.7	9.5											1980
1997	1.5	2.7	3.2	5.2												1985
2002	1.4	2.4	2.6													1990
2007	1.2	1.9														1995
2012	0.9															2000
2017																

Table S2: Number of Stroke Deaths by Age, Period, and Median Birth Cohorts in South Asia, 1990 to 2019

Median Period	Age group															Median Birth Cohort
	15-19	20-24	25-29	30-34	35-39	40-44	45-49	50-54	55-59	60-64	65-69	70-74	75-79	80-84	85-89	
Bangladesh															5995	1905
														7988	6651	1910
													9905	9350	10168	1915
												8164	9408	12762	10107	1920
											7467	8526	11180	15895	8012	1925
										9247	9933	14425	18855	16927	17155	1930
									4367	8918	11749	18801	18701	23716		1935
								4421	4639	10793	15667	19903	21959			1940
							2497	4686	5488	13078	15003	21362				1945
						2218	2775	5450	7501	13950	18057					1950
					1442	2254	3134	6614	8349	14410						1955
				1133	1529	2386	3559	7543	8588							1960
			830	1187	1702	2771	4153	7403								1965
		527	812	1238	1869	2560	3875									1970
	346	499	744	1402	1581	2645										1975
1992	312	523	763	1106	1701											1980
1997	334	555	623	1254												1985
2002	353	493	784													1990
2007	261	528														1995
2012	264															2000
2017																

Median Period	Age group															Median Birth Cohort
	15-19	20-24	25-29	30-34	35-39	40-44	45-49	50-54	55-59	60-64	65-69	70-74	75-79	80-84	85-89	
India															13278	1905
														30083	17493	1910
													54330	32314	18907	1915
												63915	65375	38924	23713	1920
											69454	76679	77546	46071	24384	1925
										61192	83704	90921	88274	48202	35595	1930
									39001	69088	90530	94781	87838	71237		1935
								24483	40486	59612	88493	100097	97029			1940
							15210	28268	39060	66407	99519	102449				1945
						8884	16853	25139	38788	76105	101233					1950
					5269	10148	17023	26673	48646	80349						1955
				3743	5902	10216	18614	32485	57361							1960
			2301	4446	7487	12490	22022	39074								1965
		2106	2727	4560	7885	13489	21590									1970
	1342	2213	2985	4605	8460	13494										1975
1992	1288	2322	2727	4513	7837											1980
1997	1426	2583	2894	4371												1985
2002	1447	2447	2226													1990
2007	1237	1780														1995
2012	809															2000
2017																

Median Period	Age group															Median Birth Cohort
	15-19	20-24	25-29	30-34	35-39	40-44	45-49	50-54	55-59	60-64	65-69	70-74	75-79	80-84	85-89	
Nepal															383	1905
														671	436	1910
													1136	771	519	1915
												1318	1320	900	667	1920
											1345	1421	1428	1067	849	1925
										1128	1325	1446	1631	1305	1038	1930
									799	1088	1279	1653	1978	1545		1935
								578	755	1044	1475	2042	2451			1940
							352	531	692	1187	1806	2455				1945
						221	324	476	777	1420	2162					1950
					118	204	280	506	900	1598						1955
				72	113	178	293	589	1014							1960
			45	70	100	186	336	646								1965
		31	43	61	100	207	364									1970
	18	32	37	58	107	209										1975
1992	20	29	36	61	107											1980
1997	18	31	37	59												1985
2002	20	36	37													1990
2007	24	38														1995
2012	26															2000
2017																

Median	Age group															Median Birth Cohort
Period	15-19	20-24	25-29	30-34	35-39	40-44	45-49	50-54	55-59	60-64	65-69	70-74	75-79	80-84	85-89	
Pakistan															4718	1905
														7670	5284	1910
													10870	7814	4940	1915
												10604	11562	7394	4706	1920
											8683	11643	11252	7199	4723	1925
										6998	10638	12086	11574	7793	5194	1930
									5159	9011	11660	12786	12669	8822		1935
								3882	6719	9865	12098	13827	13860			1940
							2448	5057	7298	10190	12773	15187				1945
						1521	3179	5446	7412	10689	13740					1950
					845	2076	3599	5722	7957	11954						1955
				633	1184	2439	3845	6176	8871							1960
			377	873	1391	2605	4040	6642								1965
		298	529	1066	1565	2871	4473									1970
	213	423	631	1201	1721	3171										1975
1992	294	521	711	1330	1939											1980
1997	354	585	789	1484												1985
2002	392	643	890													1990
2007	464	768														1995
2012	559															2000
2017																

Median Period	Age group															Median Birth Cohort
	15-19	20-24	25-29	30-34	35-39	40-44	45-49	50-54	55-59	60-64	65-69	70-74	75-79	80-84	85-89	
South Asia															24384	1905
														46428	29875	1910
													76270	50268	34548	1915
												84034	87701	60004	39212	1920
											86981	98308	101447	70264	37992	1925
										78592	105637	118922	120383	74265	59013	1930
									49344	88133	115255	128067	121242	105364		1935
								33377	52618	81341	117770	135918	135358			1940
							20515	38554	52555	90888	129140	141505				1945
						12849	23138	36522	54495	102192	135234					1950
					7677	14686	24043	39526	65870	108341						1955
				5584	8731	15223	26319	46805	75852							1960
			3554	6578	10683	18055	30557	53777								1965
		2964	4112	6927	11421	19131	30309									1970
	1921	3169	4398	7268	11872	19523										1975
1992	1914	3397	4238	7012	11587											1980
1997	2133	3754	4345	7170												1985
2002	2213	3620	3938													1990
2007	1987	3115														1995
2012	1658															2000
2017																

Table S3: Estimated Longitudinal Age and Cross-Sectional Age-Specific Trends in Stroke for the Periods 1990–1994 and 2015–2019

	Longitudinal age curve				Age-specific trend in period 1990-1994				Age-specific trend in period 2015-2019			
	Curve Equation	R square	RR_{10}	RR_{90-94}	Curve Equation	R square	RR_{10}	RR_{90-94}	Curve Equation	R square	RR_{10}	RR_{90-94}
Bangladesh	$y=0.76*e^{0.10*mean\_age}$	0.99	2.61	195.3	$y=0.78*e^{0.10*mean\_age}$	0.99	2.72	244.7	$y=0.41*e^{0.11*mean\_age}$	0.99	2.92	365.6
India	$y=0.43*e^{0.09*mean\_age}$	0.98	2.46	142.0	$y=0.31*e^{0.11*mean\_age}$	0.99	2.89	346.0	$y=0.19*e^{0.11*mean\_age}$	0.98	2.92	363.6
Nepal	$y=0.37*e^{0.09*mean\_age}$	0.99	2.57	180.8	$y=0.27*e^{0.11*mean\_age}$	0.98	2.95	384.1	$y=0.13*e^{0.11*mean\_age}$	0.99	3.14	537.3
Pakistan	$y=0.56*e^{0.10*mean\_age}$	0.99	2.61	197.5	$y=0.44*e^{0.10*mean\_age}$	0.99	2.84	308.3	$y=0.57*e^{0.10*mean\_age}$	0.99	2.69	232.9
South Asia	$y=0.47*e^{0.09*mean\_age}$	0.98	2.51	157.6	$y=0.36*e^{0.11*mean\_age}$	0.99	2.88	336.6	$y=0.26*e^{0.11*mean\_age}$	0.99	2.87	327.5

Table S4: Annual Percentage Change for Stroke Mortality Overall and in Each Age Group Across Four South Asian Countries from 1990 to 2019

Age groups	Bangladesh	India	Nepal	Pakistan
**Both sexes**				
15-19	-1.1(-3.9,1.9)	-3.6(-6.3,-0.8)	-1.2(-2.6,0.3)	0.9(0.3,1.5)
20-24	-1.1(-3.0,0.7)	-2.4(-4.0,-0.7)	-1.8(-2.8,-0.8)	0.6(0.2,1.0)
25-29	-1.5(-2.9,-0.1)	-1.8(-3.0,-0.5)	-2.4(-3.2,-1.5)	0.3(0.0,0.7)
30-34	-1.8(-2.9,-0.7)	-1.3(-2.3,-0.3)	-2.6(-3.2,-1.9)	0.0(-0.3,0.3)
35-39	-1.7(-2.6,-0.8)	-0.9(-1.7,-0.1)	-2.5(-3.0,-2.0)	-0.3(-0.6,-0.1)
40-44	-1.6(-2.3,-0.8)	-0.5(-1.1,0.1)	-2.3(-2.7,-1.9)	-0.6(-0.8,-0.4)
45-49	-1.3(-1.9,-0.7)	-0.8(-1.2,-0.3)	-2.1(-2.4,-1.8)	-0.8(-1.0,-0.7)
50-54	-1.2(-1.7,-0.7)	-0.9(-1.2,-0.5)	-1.9(-2.1,-1.7)	-0.9(-1.0,-0.7)
55-59	-1.1(-1.5,-0.7)	-1.4(-1.7,-1.1)	-1.7(-1.9,-1.6)	-0.8(-0.9,-0.7)
60-64	-1.0(-1.3,-0.6)	-1.7(-2.0,-1.4)	-1.6(-1.7,-1.4)	-0.7(-0.8,-0.6)
65-69	-0.8(-1.1,-0.4)	-2.0(-2.2,-1.7)	-1.4(-1.5,-1.2)	-0.6(-0.7,-0.5)
70-74	-0.8(-1.1,-0.5)	-2.1(-2.3,-1.8)	-1.1(-1.3,-1.0)	-0.5(-0.6,-0.4)
75-79	-0.6(-1.0,-0.3)	-2.3(-2.6,-2.0)	-0.9(-1.0,-0.7)	-0.5(-0.6,-0.4)
80-84	-0.1(-0.5,0.3)	-2.2(-2.6,-1.9)	-0.6(-0.8,-0.4)	-0.6(-0.7,-0.4)
85-89	0.5(-0.1,1.1)	-2.3(-3.0,-1.6)	-0.2(-0.6,0.1)	-0.6(-0.8,-0.5)
**Overall**	-1.1(-1.5,-0.8)	-1.5(-1.8,-1.2)	-1.7(-1.9,-1.6)	-0.5(-0.5,-0.4)
**Women**				
15-19	-1.3(-4.5,2.1)	-3.9(-7.2,-0.5)	-3.3(-6.1,-0.5)	0.6(0.0,1.1)
20-24	-2.0(-4.4,0.4)	-3.0(-5.0,-0.9)	-3.3(-5.0,-1.5)	0.3(-0.2,0.7)
25-29	-2.6(-4.5,-0.7)	-2.6(-4.2,-1.0)	-3.3(-4.6,-2.0)	-0.1(-0.5,0.2)
30-34	-2.8(-4.3,-1.3)	-1.9(-3.1,-0.6)	-3.3(-4.2,-2.3)	-0.4(-0.7,-0.1)
35-39	-2.5(-3.7,-1.2)	-1.2(-2.2,-0.2)	-3.1(-3.8,-2.4)	-0.7(-1.0,-0.5)
40-44	-2.2(-3.2,-1.2)	-0.7(-1.5,0.1)	-3.0(-3.6,-2.5)	-1.0(-1.1,-0.8)
45-49	-1.8(-2.5,-1.0)	-0.8(-1.4,-0.2)	-3.0(-3.4,-2.6)	-1.1(-1.2,-0.9)
50-54	-1.5(-2.1,-0.8)	-0.7(-1.2,-0.2)	-3.0(-3.3,-2.6)	-1.1(-1.2,-0.9)
55-59	-1.3(-1.9,-0.8)	-1.5(-1.9,-1.1)	-2.8(-3.1,-2.5)	-0.9(-1.0,-0.8)
60-64	-1.3(-1.7,-0.8)	-1.7(-2.0,-1.3)	-2.6(-2.8,-2.4)	-0.8(-0.8,-0.7)
65-69	-0.9(-1.3,-0.5)	-1.9(-2.2,-1.6)	-2.3(-2.5,-2.1)	-0.6(-0.7,-0.5)
70-74	-0.9(-1.3,-0.5)	-2.1(-2.4,-1.8)	-1.8(-2.0,-1.6)	-0.5(-0.6,-0.4)
75-79	-0.6(-1.0,-0.2)	-2.4(-2.8,-2.1)	-1.3(-1.6,-1.1)	-0.4(-0.5,-0.3)
80-84	-0.1(-0.6,0.4)	-2.3(-2.8,-1.8)	-0.8(-1.1,-0.5)	-0.3(-0.4,-0.2)
85-89	0.6(-0.1,1.3)	-2.3(-3.2,-1.5)	-0.3(-0.8,0.1)	-0.3(-0.4,-0.1)
**Overall**	-1.5(-2.0,-1.1)	-1.7(-2.1,-1.3)	-2.6(-2.9,-2.3)	-0.6 (-0.7,-0.5)
**Men**				
15-19	-1.2(-4.5,2.2)	-3.2(-6.0,-0.3)	-0.1(-1.8,1.7)	1.2(0.6,1.8)
20-24	-0.7(-2.6,1.2)	-1.7(-3.4,-0.1)	-0.8(-2.1,0.5)	1.0(0.6,1.4)
25-29	-0.9(-2.3,0.5)	-1.1(-2.3,0.2)	-1.5(-2.6,-0.4)	0.7(0.4,1.1)
30-34	-1.1(-2.2,0.0)	-0.8(-1.8,0.1)	-1.9(-2.8,-1.0)	0.4(0.1,0.7)
35-39	-1.0(-1.9,-0.1)	-0.6(-1.3,0.1)	-1.8(-2.5,-1.1)	0.1(-0.2,0.3)

40-44	-1.0(-1.7,-0.2)	-0.4(-0.9,0.2)	-1.6(-2.1,-1.1)	-0.3(-0.5,-0.1)
45-49	-0.9(-1.5,-0.3)	-0.6(-1.1,-0.2)	-1.3(-1.7,-0.9)	-0.5(-0.7,-0.4)
50-54	-1.0(-1.5,-0.4)	-0.9(-1.2,-0.5)	-1.0(-1.3,-0.7)	-0.6(-0.8,-0.5)
55-59	-0.9(-1.4,-0.4)	-1.3(-1.6,-1.0)	-0.8(-1.1,-0.6)	-0.6(-0.8,-0.5)
60-64	-0.7(-1.1,-0.3)	-1.7(-2.0,-1.4)	-0.7(-0.9,-0.5)	-0.6(-0.7,-0.5)
65-69	-0.7(-1.0,-0.3)	-2.0(-2.2,-1.7)	-0.6(-0.8,-0.5)	-0.6(-0.7,-0.5)
70-74	-0.8(-1.1,-0.4)	-2.0(-2.3,-1.8)	-0.6(-0.8,-0.4)	-0.7(-0.7,-0.6)
75-79	-0.7(-1.0,-0.3)	-2.2(-2.5,-1.9)	-0.5(-0.7,-0.3)	-0.8(-0.9,-0.7)
80-84	-0.1(-0.6,0.3)	-2.2(-2.6,-1.8)	-0.4(-0.7,-0.1)	-1.0(-1.1,-0.8)
85-89	0.4(-0.3,1.1)	-2.3(-3.0,-1.6)	-0.1(-0.6,0.3)	-1.2(-1.3,-1.0)
**Overall**	-0.8(-1.2,-0.4)	-1.3(-1.6,-1.1)	-1.0(-1.3,-0.8)	-0.3(-0.4,-0.2)

Table S5: Fitted Longitudinal Age Effects of Stroke Mortality (per 100,000 person-years) and the Corresponding 95% CIs

Age groups	Bangladesh	India	Nepal	Pakistan
**Both sexes**				
15-19	3.9(2.6,5.9)	1.8(1.3,2.6)	1.7(1.4,2.2)	2.3(2.1,2.5)
20-24	6.7(4.8,9.3)	3.1(2.4,4.1)	2.9(2.4,3.5)	4.1(3.7,4.4)
25-29	10.1(7.8,13.2)	3.7(2.9,4.7)	3.7(3.2,4.4)	6.0(5.6,6.4)
30-34	17.4(14.1,21.6)	6.2(5.1,7.4)	6.4(5.7,7.2)	12.2(11.5,12.9)
35-39	25.8(21.6,30.9)	10.7(9.2,12.4)	11.1(10.1,12.2)	19.0(18.1,19.9)
40-44	43.6(37.7,50.3)	19.0(16.9,21.4)	21.7(20.2,23.2)	37.4(36.0,38.7)
45-49	68.2(60.3,77.3)	35.7(32.5,39.2)	37.3(35.3,39.4)	64.2(62.4,66.1)
50-54	144.7(131.2,159.6)	67.9(62.9,73.4)	68.5(65.5,71.6)	117.4(114.6,120.3)
55-59	183.6(167.7,201.1)	116.1(108.7,124.1)	110.6(106.4,114.9)	189.7(185.7,193.8)
60-64	392.6(363.6,424.0)	197.0(186.0,208.6)	187.7(181.5,194.1)	322.9(316.6,329.4)
65-69	540.4(501.1,582.8)	304.3(287.9,321.7)	284.7(275.8,293.9)	495.4(485.8,505.2)
70-74	809.7(741.5,884.2)	402.5(377.3,429.4)	430.2(414.4,446.6)	743.5(726.9,760.5)
75-79	1,181.2(1,077.0,1,295.4)	547.4(511.3,586.1)	644.6(619.9,670.2)	1,085.0(1,059.5,1,111.2)
80-84	2,095.8(1,902.5,2,308.7)	580.6(537.2,627.5)	735.3(704.2,767.8)	1,257.6(1,224.5,1,291.6)
85-89	3,409.3(3,062.5,3,795.5)	748.8(680.6,823.8)	1,069.7(1,018.0,1,124.1)	1,783.5(1,729.1,1,839.6)
**Men**				
15-19	3.5(2.2,5.5)	1.6(1.1,2.3)	1.8(1.3,2.4)	1.9(1.7,2.1)
20-24	8.0(5.8,11.1)	2.7(2.0,3.5)	3.1(2.4,3.9)	3.9(3.6,4.3)
25-29	12.8(9.9,16.7)	3.4(2.7,4.3)	3.5(2.9,4.4)	5.6(5.2,6.0)
30-34	22.6(18.3,27.9)	6.9(5.8,8.3)	6.5(5.6,7.7)	10.7(10.1,11.3)
35-39	31.8(26.6,38.1)	12.1(10.5,14.0)	11.1(9.8,12.6)	18.5(17.6,19.4)
40-44	49.1(42.3,57.0)	21.8(19.5,24.4)	21.6(19.6,23.7)	34.0(32.7,35.3)

45-49	80.3(70.7,91.3)	40.2(36.8,43.9)	36.0(33.4,38.9)	57.5(55.7,59.3)
50-54	161.1(145.4,178.4)	78.9(73.4,84.7)	77.1(72.7,81.8)	117.3(114.4,120.3)
55-59	204.7(186.1,225.1)	133.0(124.9,141.6)	128.1(121.8,134.8)	182.0(177.9,186.1)
60-64	444.4(409.9,481.9)	217.6(205.8,230.1)	224.9(215.1,235.1)	308.2(301.9,314.7)
65-69	578.7(533.8,627.3)	334.9(317.2,353.6)	364.0(349.2,379.4)	488.7(478.8,498.8)
70-74	815.1(741.7,895.8)	439.2(412.3,467.9)	545.9(519.5,573.5)	699.8(683.4,716.6)
75-79	1,164.6(1,054.1,1,286.6)	585.7(547.7,626.4)	811.1(770.2,854.3)	978.3(954.0,1,003.2)
80-84	2,125.0(1,914.7,2,358.5)	603.5(558.3,652.3)	993.1(937.4,1,052.0)	1,089.8(1,059.2,1,121.3)
85-89	3,328.1(2,961.3,3,740.4)	772.8(701.1,851.8)	1,526.7(1,428.5,1,631.6)	1,549.0(1,498.3,1,601.3)
**Women**				
15-19	4.7(2.9,7.6)	2.2(1.4,3.4)	1.5(1.0,2.3)	2.8(2.6,3.1)
20-24	5.3(3.4,8.1)	3.8(2.7,5.2)	2.7(2.0,3.6)	4.1(3.8,4.5)
25-29	7.2(5.0,10.2)	4.0(3.0,5.3)	4.1(3.2,5.1)	6.4(6.0,6.9)
30-34	11.1(8.3,14.9)	5.3(4.1,6.8)	6.3(5.2,7.5)	13.8(13.1,14.6)
35-39	18.2(14.3,23.2)	9.0(7.4,11.0)	11.2(9.8,12.9)	19.3(18.4,20.3)
40-44	36.7(30.6,44.1)	15.8(13.5,18.5)	21.9(19.8,24.3)	41.0(39.5,42.5)
45-49	53.8(45.9,63.0)	30.5(26.9,34.5)	38.9(35.9,42.2)	71.4(69.3,73.5)
50-54	125.2(110.8,141.5)	55.8(50.2,62.0)	59.2(55.3,63.4)	117.2(114.3,120.1)
55-59	159.3(142.4,178.3)	98.6(90.3,107.5)	92.7(87.3,98.3)	198.4(194.0,202.9)
60-64	332.6(302.6,365.6)	177.0(164.4,190.6)	152.0(144.3,160.1)	339.8(332.9,346.8)
65-69	495.4(452.3,542.6)	275.9(256.9,296.2)	212.6(202.3,223.4)	502.2(492.2,512.5)
70-74	799.9(719.9,888.7)	370.9(341.4,403.1)	325.1(306.9,344.3)	789.0(771.0,807.5)
75-79	1,194.6(1,070.3,1,333.3)	518.4(475.3,565.5)	493.7(465.3,523.9)	1,193.0(1,164.4,1,222.3)
80-84	2,048.3(1,825.1,2,298.9)	566.8(514.3,624.6)	522.2(489.1,557.7)	1,424.4(1,386.6,1,463.3)
85-89	3,480.0(3,066.7,3,949.1)	737.7(656.0,829.5)	725.0(672.9,781.1)	2,000.7(1,939.4,2,064.0)

Table S6: Relative Risk of Each Period Compared with the Reference (2000 – 2004) and the Corresponding 95% CIs

**Periods**	**Bangladesh**	**India**	**Nepal**	**Pakistan**
**Both sexes**				
1990-1994	1.00(0.92,1.08)	1.07(1.01,1.14)	1.45(1.40,1.50)	0.90(0.88,0.91)
1995-1999	0.93(0.86,0.99)	1.08(1.03,1.14)	1.22(1.18,1.26)	1.01(1.00,1.03)
2000-2004	Reference period	Reference period	Reference period	Reference period
2005-2009	1.05(0.99,1.12)	0.90(0.86,0.95)	0.93(0.90,0.96)	0.94(0.92,0.95)
2010-2014	0.81(0.75,0.86)	0.82(0.78,0.86)	0.93(0.90,0.96)	0.88(0.87,0.90)
2015-2019	0.73(0.68,0.78)	0.76(0.72,0.80)	0.93(0.90,0.96)	0.84(0.82,0.85)
**Men**				
1990-1994	0.99(0.91,1.08)	1.05(0.99,1.11)	1.32(1.26,1.38)	0.86(0.84,0.88)
1995-1999	0.96(0.89,1.04)	1.08(1.03,1.14)	1.18(1.13,1.23)	1.00(0.99,1.02)
2000-2004	Reference period	Reference period	Reference period	Reference period
2005-2009	1.14(1.07,1.22)	0.92(0.88,0.97)	0.97(0.93,1.01)	0.95(0.94,0.97)
2010-2014	0.83(0.77,0.89)	0.86(0.81,0.90)	1.00(0.96,1.05)	0.89(0.88,0.91)
2015-2019	0.79(0.74,0.85)	0.76(0.72,0.81)	1.02(0.98,1.07)	0.83(0.82,0.85)
**Women**				
1990-1994	1.01(0.92,1.11)	1.09(1.01,1.18)	1.61(1.53,1.70)	0.93(0.92,0.95)
1995-1999	0.88(0.80,0.95)	1.08(1.01,1.15)	1.27(1.21,1.33)	1.02(1.00,1.04)
2000-2004	Reference period	Reference period	Reference period	Reference period
2005-2009	0.94(0.87,1.01)	0.88(0.83,0.94)	0.89(0.85,0.93)	0.92(0.91,0.94)
2010-2014	0.77(0.71,0.83)	0.78(0.73,0.83)	0.85(0.81,0.89)	0.87(0.86,0.89)
2015-2019	0.65(0.59,0.71)	0.75(0.70,0.81)	0.83(0.78,0.87)	0.84(0.82,0.85)

Table S7: Relative Risk of Each Cohort Compared with the Reference (Cohort 1955–1959) and the Corresponding 95% CIs

**Cohorts**	**Bangladesh**	**India**	**Nepal**	**Pakistan**
**Both sexes**				
1905-1909	1.04(0.84,1.28)	2.66(2.11,3.35)	1.43(1.28,1.61)	1.34(1.27,1.42)
1910-1914	1.09(0.94,1.27)	2.32(2.03,2.65)	1.47(1.37,1.58)	1.28(1.23,1.33)
1915-1919	1.28(1.14,1.44)	2.12(1.92,2.34)	1.48(1.40,1.56)	1.23(1.19,1.27)
1920-1924	1.30(1.16,1.45)	1.89(1.74,2.05)	1.45(1.38,1.52)	1.20(1.16,1.23)
1925-1929	1.22(1.10,1.35)	1.66(1.54,1.79)	1.41(1.36,1.47)	1.16(1.13,1.19)
1930-1934	1.16(1.06,1.27)	1.47(1.37,1.57)	1.36(1.31,1.41)	1.14(1.11,1.16)
1935-1939	1.14(1.04,1.24)	1.34(1.26,1.43)	1.27(1.22,1.32)	1.11(1.08,1.13)
1940-1944	1.13(1.03,1.23)	1.18(1.11,1.26)	1.18(1.14,1.23)	1.08(1.05,1.10)
1945-1949	1.03(0.95,1.13)	1.13(1.06,1.20)	1.09(1.05,1.13)	1.04(1.02,1.07)
1950-1954	Reference cohort	Reference cohort	Reference cohort	Reference cohort
1955-1959	0.91(0.82,1.00)	0.97(0.90,1.04)	0.91(0.87,0.95)	0.96(0.93,0.98)
1960-1964	0.88(0.79,0.99)	0.96(0.87,1.04)	0.82(0.78,0.86)	0.91(0.88,0.94)
1965-1969	0.82(0.72,0.94)	0.97(0.87,1.08)	0.73(0.68,0.78)	0.88(0.85,0.91)
1970-1974	0.74(0.62,0.88)	0.88(0.77,1.02)	0.65(0.59,0.70)	0.86(0.83,0.90)
1975-1979	0.66(0.54,0.82)	0.88(0.74,1.04)	0.56(0.50,0.62)	0.86(0.82,0.91)
1980-1984	0.61(0.47,0.79)	0.76(0.61,0.95)	0.49(0.42,0.56)	0.88(0.83,0.94)
1985-1989	0.57(0.41,0.79)	0.70(0.53,0.93)	0.43(0.35,0.52)	0.90(0.84,0.98)
1990-1994	0.57(0.38,0.86)	0.61(0.42,0.89)	0.42(0.33,0.53)	0.95(0.86,1.04)
1995-1999	0.55(0.31,0.97)	0.49(0.29,0.81)	0.42(0.31,0.56)	1.01(0.89,1.13)
2000-2004	0.48(0.19,1.24)	0.33(0.13,0.84)	0.41(0.26,0.65)	1.08(0.91,1.28)
**Men**				
1905-1909	1.02(0.81,1.29)	2.65(2.10,3.36)	1.17(1.01,1.37)	1.51(1.42,1.60)

1910-1914	1.07(0.91,1.25)	2.33(2.03,2.66)	1.22(1.10,1.34)	1.40(1.35,1.46)
1915-1919	1.24(1.09,1.41)	2.09(1.89,2.31)	1.22(1.13,1.31)	1.31(1.26,1.35)
1920-1924	1.26(1.12,1.42)	1.86(1.71,2.02)	1.19(1.12,1.27)	1.23(1.19,1.27)
1925-1929	1.20(1.07,1.34)	1.65(1.54,1.77)	1.17(1.11,1.24)	1.17(1.14,1.20)
1930-1934	1.10(1.00,1.22)	1.48(1.39,1.58)	1.15(1.09,1.21)	1.13(1.10,1.16)
1935-1939	1.10(1.00,1.21)	1.36(1.28,1.45)	1.11(1.06,1.17)	1.10(1.07,1.13)
1940-1944	1.08(0.99,1.18)	1.20(1.12,1.27)	1.08(1.03,1.13)	1.07(1.04,1.09)
1945-1949	1.02(0.94,1.12)	1.13(1.06,1.20)	1.04(0.99,1.09)	1.04(1.02,1.06)
1950-1954	Reference cohort	Reference cohort	Reference cohort	Reference cohort
1955-1959	0.92(0.83,1.02)	0.99(0.92,1.07)	0.95(0.90,1.01)	0.97(0.94,0.99)
1960-1964	0.89(0.79,1.01)	1.00(0.92,1.08)	0.90(0.84,0.96)	0.94(0.91,0.96)
1965-1969	0.85(0.74,0.98)	0.95(0.86,1.05)	0.83(0.76,0.90)	0.92(0.89,0.95)
1970-1974	0.83(0.69,0.99)	0.93(0.81,1.06)	0.75(0.67,0.84)	0.92(0.88,0.96)
1975-1979	0.76(0.61,0.94)	0.93(0.79,1.10)	0.67(0.58,0.78)	0.94(0.89,1.00)
1980-1984	0.71(0.54,0.92)	0.84(0.68,1.04)	0.61(0.50,0.74)	0.98(0.92,1.05)
1985-1989	0.68(0.50,0.94)	0.80(0.61,1.05)	0.57(0.44,0.73)	1.03(0.95,1.12)
1990-1994	0.71(0.47,1.06)	0.72(0.49,1.04)	0.59(0.43,0.80)	1.10(1.00,1.21)
1995-1999	0.68(0.38,1.22)	0.59(0.36,0.99)	0.62(0.43,0.89)	1.17(1.03,1.32)
2000-2004	0.51(0.17,1.55)	0.38(0.15,0.97)	0.64(0.38,1.10)	1.28(1.07,1.54)
**Women**				
1905-1909	1.06(0.84,1.35)	2.61(1.96,3.47)	1.82(1.54,2.14)	1.23(1.16,1.31)
1910-1914	1.14(0.96,1.35)	2.28(1.94,2.69)	1.84(1.66,2.05)	1.20(1.16,1.25)
1915-1919	1.34(1.17,1.55)	2.12(1.88,2.40)	1.86(1.71,2.02)	1.19(1.16,1.23)
1920-1924	1.35(1.19,1.54)	1.89(1.70,2.10)	1.82(1.70,1.95)	1.19(1.15,1.22)
1925-1929	1.26(1.11,1.42)	1.64(1.50,1.81)	1.76(1.66,1.88)	1.17(1.14,1.20)

1930-1934	1.23(1.10,1.37)	1.43(1.31,1.56)	1.66(1.56,1.76)	1.15(1.12,1.17)
1935-1939	1.18(1.06,1.31)	1.32(1.21,1.43)	1.49(1.40,1.57)	1.12(1.09,1.15)
1940-1944	1.18(1.07,1.31)	1.15(1.06,1.25)	1.32(1.25,1.40)	1.09(1.06,1.11)
1945-1949	1.05(0.94,1.16)	1.12(1.03,1.22)	1.16(1.09,1.22)	1.05(1.03,1.08)
1950-1954	Reference cohort	Reference cohort	Reference cohort	Reference cohort
1955-1959	0.90(0.79,1.01)	0.94(0.85,1.04)	0.85(0.80,0.91)	0.95(0.92,0.97)
1960-1964	0.88(0.76,1.01)	0.91(0.80,1.02)	0.73(0.67,0.79)	0.89(0.86,0.91)
1965-1969	0.80(0.68,0.95)	1.02(0.89,1.18)	0.63(0.57,0.69)	0.84(0.81,0.87)
1970-1974	0.65(0.51,0.81)	0.84(0.70,1.01)	0.55(0.48,0.62)	0.81(0.77,0.84)
1975-1979	0.57(0.43,0.75)	0.82(0.65,1.04)	0.46(0.39,0.54)	0.79(0.75,0.84)
1980-1984	0.51(0.36,0.73)	0.67(0.50,0.91)	0.38(0.31,0.48)	0.79(0.74,0.85)
1985-1989	0.43(0.27,0.68)	0.61(0.42,0.88)	0.32(0.24,0.43)	0.79(0.73,0.86)
1990-1994	0.41(0.23,0.72)	0.51(0.32,0.82)	0.28(0.19,0.41)	0.82(0.74,0.90)
1995-1999	0.40(0.19,0.84)	0.39(0.21,0.74)	0.24(0.14,0.41)	0.87(0.77,0.98)
2000-2004	0.43(0.15,1.19)	0.29(0.09,0.89)	0.19(0.08,0.48)	0.91(0.77,1.08)
